# Season of birth and handedness in Serbian high school students

**DOI:** 10.1186/1744-859X-7-2

**Published:** 2008-01-30

**Authors:** Sanja Milenković, Daniel Rock, Milan Dragović, Aleksandar Janca

**Affiliations:** 1 Institute for Hygiene and Medical Ecology, School of Medicine, University of Belgrade, Serbia; 2 School of Psychiatry and Clinical Neurosciences, University of Western Australia, Australia; 3 Centre for Clinical Research in Neuropsychiatry, Graylands Hospital, Western Australia, Australia

## Abstract

**Background:**

Although behavioural dominance of the right hand in humans is likely to be under genetic control, departures from this population norm, i.e. left- or non-right-handedness, are believed to be influenced by environmental factors. Among many such environmental factors including, for example, low birth weight, testosterone level, and maternal age at birth, season of birth has occasionally been investigated. The overall empirical evidence for the season of birth effect is mixed.

**Methods:**

We have investigated the effect of season of birth in an epidemiologically robust sample of randomly selected young people (n = 977), all born in the same year. A Kolmogorov-Smirnov type statistical test was used to determine season of birth.

**Results:**

Neither the right-handed nor the non-right-handed groups demonstrated birth asymmetry relative to the normal population birth distribution. There was no between-group difference in the seasonal distribution of birth when comparing the right-handed to the non-right-handed groups.

**Conclusion:**

The present study failed to provide support for a season of birth effect on atypical lateralisation of handedness in humans.

## Background

Functional dominance of the right hand is the norm across different populations, various geographical regions, and diverse cultures, with approximately 90% of humans exhibiting clear dominance of the right side of the body. This behavioural characteristic is considered as uniquely human, as there is no other species that displays such a large behavioural asymmetry at the population level. It is also widely accepted that this behavioural feature emerged at some point during the hominid evolution, and that this feature preceded the evolution of another uniquely human feature – language, and in particular, speech as its central component [1].

The transmission of handedness over many generations of humans is widely believed to be under genetic control [2-4], rather than resulting from learning. Converging lines of evidence provide support for the genetic hypothesis, including imaging studies on twins [5], meta-analysis of handedness in twins [6], and molecular genetic studies [7,8]. To date, however, no gene for handedness has been identified. Genetic models of handedness [2,3] argue that the functional advantage of the right hand originates from a purely genetic effect, while left-handedness is a consequence of a random shift in hand dominance. Theory suggests that in individuals without the genetic disposition, both cerebral and hand dominance are randomly assigned. An implication of these models is that left-sided behavioural dominance is a benign genetic consequence, but not a pathology acquired during early brain development. Moreover, these models also argue that left-handedness may be beneficial. For example, there is empirical support for the notion that left-handers are somewhat better in visuospatial and visuomotor abilities than right-handers This may explain why left-handers are overrepresented in some groups with high demand on spatial skills such as architects [9], tennis players and cricketers [10], and musicians [11]. In contrast to purely genetic models, the shift away from the "default" right-handedness has occasionally been labelled as "anomalous" (e.g. [12]), "alinormal" (e.g. [13]), or "atypical" (e.g. [14]). The increased prevalence of left-handedness in populations with some medical conditions (e.g. Rett syndrome, schizophrenia, autism) is believed to originate from pathological processes that either overpower or disrupt the genetics of hand dominance.

The environmental factors believed to provide structural brain substrate for left-handedness include birth difficulties [15], prenatal ultrasound [16], maternal smoking during pregnancy [17], low birth weight [18,19], diffuse brain damage [20], and testosterone level during early development [12]. Another factor that has occasionally been considered as "trigger" for atypical lateralisation of hand preferences is season of birth. That season of birth can be a serious risk factor has already been established for various conditions, including brain tumours [21], proneness to road accidents [22], and schizophrenia [23]. How season of birth may exert an influence on cerebral lateralisation is less clear. Season of birth may be conceptualised as a portmanteau term covering various environmental variables such as prenatal exposure to various hormones (e.g. testosterone), incidence of diseases, nutrition, and reproductive activity in humans. For obvious reasons, direct investigations between these factors and behavioural lateralisation are not always feasible. It has therefore been hypothesised that variation in the incidence of viral infections [24,25] and prenatal exposure to testosterone [12] may be responsible for sinistral developmental trajectory. Similar to all other environmental factors responsible for left-handedness, the empirical evidence for the season of birth effect is indirect, speculative and, at best, mixed. In this paper we describe the effect of season of birth on atypical lateralisation of hand preferences in a large sample of students all born in the same year.

## Methods

### Aim of the study

The aim of this study is to examine season of birth as a risk factor for hand preference. We used a population-representative random sample of high school students, grouped according to hand preference. We compared their birth distribution with the corresponding age-matched birth distribution in the general population.

### Participants

A total of 1 224 high school students participated in this study. The sample comprised all year 9 students (mean age = 15.0 years, SD = 0.4) from six randomly selected high schools in Belgrade, Serbia. Students born in 1989 and 1991 and those with a missing date of birth were excluded from analysis, leaving thus 977 students born in a single year (1990). The reduced sample comprised 457 males (9.4% left-hand writers) and 520 females (5.4% left-hand writers). Whole population birth data were provided by the Statistical Office of the Republic of Serbia.

### Assessment

Handedness was assessed by the Edinburgh Handedness Inventory [26]. This inventory comprises 10 items for hand preference and two additional laterality preferences (eye and foot) that were excluded from analysis. On each item participants indicated their hand preference in the following range: strong (++), less strong (+), to indifferent (+/+). Laterality quotients ranging from -100 (left-handedness) to +100 (right-handedness) were computed for each subject in the study, using the standard expression LQ = (R-L)/(R+L)*100. Subjects (n = 247) with laterality quotients in the range -100 to +50 were considered as clearly not right-hand dominant, while the remaining subjects (from +51 to +100) were classified as consistent right-handers (n = 730). This classification of subjects is based on neurological and neurobehavioural research [5,9,12] that supports the notion of a taxonic structure of hand preferences, i.e. strong right and non-right. To separate strong right-handers from non-right-handers, a conservative threshold for determination of non-right-handedness was selected [27]. The small number of exclusive left-handers in the sample precluded the analysis of these as a separate birth group.

### Determination of season of birth

Date of birth was collapsed into a single 12-month frequency series. Season of birth was determined using a Kolmogorov-Smirnov type statistical test [28]. This test has been proposed as a more specific test of the curvilinear variation that is characteristic of birth series and has been used in other seasonality studies (e.g. [29-31]). Since this method compares the cumulative proportional difference curves between two contemporaneous time series, it can accommodate the variable population of risk approach. Variable population of risk adjustment was achieved by comparing the birth distribution of "handedness" groups with whole population data, again collapsed into a single 12-month frequency series. All of the "handedness" group are a single birth cohort, born in the same year. National population live birth data for the same birth year was used as the index, or expected birth distribution. Using this approach, we can determine whether there is a significant difference in the frequency distribution of birth months between the handedness sub-groups and the general population variation of births by month. Data were adjusted to a standard 31-day month to eliminate the "calendar effect" [32].

## Results

**Figure 1:**
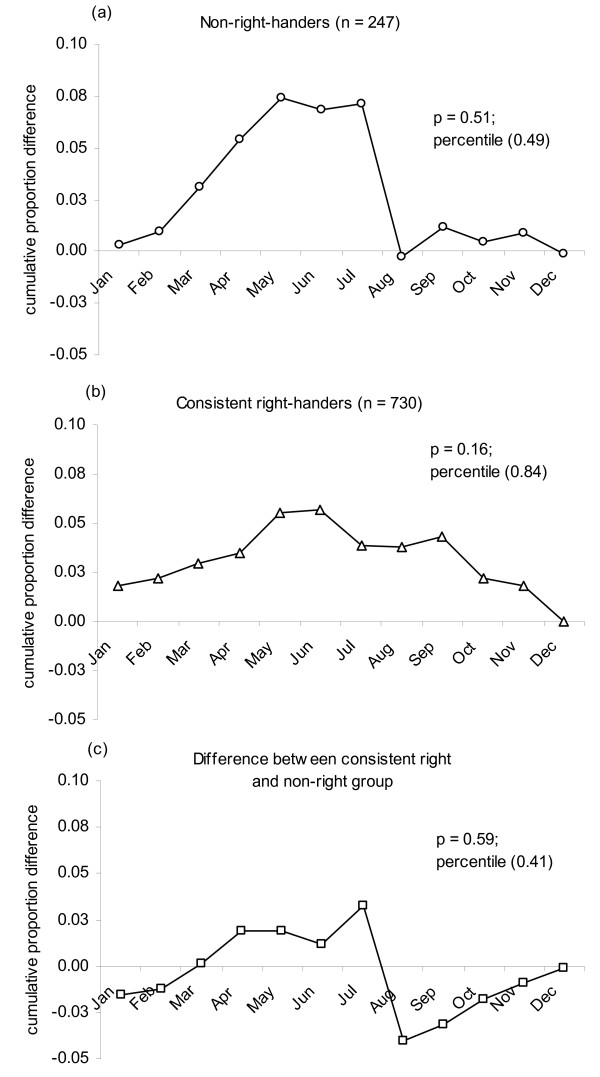
**Cumulative proportional differences**. Cumulative proportional difference between the birth distribution for the dependent samples, (a) non-right-handed and (b) right-handed, and the normal whole population distribution of births for the same birth year (1990) are shown. (c) Cumulative proportional difference between the two dependent birth distributions.

Neither the right-handed nor the non-right-handed groups showed a birth asymmetry relative to the normal population birth distribution. Furthermore, there was no between-groups difference in the seasonal distribution of birth comparing the right-handed to the non-right-handed groups. Figure 1a–c shows the cumulative proportional difference in the two sub-samples compared with the general population (a, b) and with each other (c).

## Discussion

The results of the present study do not support an association between the distribution of handedness and season of birth in young people.

It is worth noting that all of our analyses are based on a sample of subjects who were all born in a single year and compared with whole of population birth distribution for the same year. As far as we are aware, this is the first study to use this procedure within a variable population at risk model. Most season of birth studies create a composite reference population, summing the different normal population yearly birth distributions for the age range of the dependent group [33]. In such a situation it can be difficult to definitively conclude that any seasonal difference between two composite birth distributions is related to the dependent sample, and is not merely an artefact associated with the "constructed" reference population. Furthermore, the sample comprised a randomly selected subset of all school-age children from Belgrade. Again, this approach has not been used previously to study season of birth effects in handedness. Others, for well-understood reasons, tend to use convenience samples. The effect such methodological differences may have has not been systematically studied in the seasonality literature, however, the advantages of random sampling, *per se*, have been well described (e.g. [34]).

Although there is some evidence for a season of birth effect on human handedness, the empirical data are inconclusive as there are studies showing contradictory results. For example, several published studies [24,25,35,36] have suggested that distribution of birth is different in left-handers than in right-handers, whereas some studies reported a gender specific association (e.g. [25,37]). By contrast, quite a few studies [38-40], including some that have re-examined previously published results (e.g. [41]), failed to confirm the pathogenic effect of season of birth on atypical hand dominance. The lack of empirical consistency of findings makes this factor, which potentially may explain a certain proportion of variation in human handedness, remain obscure.

In conclusion, we found no evidence that season of birth possess an aetiological relevance for developing atypical lateralisation of hand preferences.

## Competing interests

The author(s) declare that they have no competing interests.

## Authors' contributions

The authors all contributed equally to the manuscript, were all involved in the drafting of the manuscript and gave final approval on the manuscript.
